# Application of PCR methods to evaluate *EGFR*, *KRAS* and *BRAF* mutations in a small number of tumor cells in cytological material from lung cancer patients

**DOI:** 10.3892/or.2013.2579

**Published:** 2013-07-01

**Authors:** MARZENA ANNA LEWANDOWSKA, WOJCIECH JÓŹWICKI, CEZARY JOCHYMSKI, JANUSZ KOWALEWSKI

**Affiliations:** 1 Molecular Oncology and Genetics Unit, Department of Tumor Pathology and Pathomorphology, Franciszek Lukaszczyk Oncology Center, Bydgoszcz, Poland; 2 Department of Thoracic Surgery and Tumors, Ludwik Rydygier Collegium Medicum, Bydgoszcz, Nicolaus Copernicus University, Torun, Poland; 3 Department of Tumor Pathology and Pathomorphology, Ludwik Rydygier Collegium Medicum, Bydgoszcz, Nicolaus Copernicus University, Torun, Poland; 4 Department of Thoracic Surgery and Tumors, Franciszek Lukaszczyk Oncology Center, Bydgoszcz, Poland

**Keywords:** molecular cytopathology, molecular diagnostics, personalized medicine, *EGFR*, *KRAS*, *BRAF*

## Abstract

The epidermal growth factor receptor (EGFR*)* mutation status in the tyrosine kinase domain is known to be a predictor of the response to gefitinib or erlotinib in lung cancer; thus, a non-surgical procedure of tumor specimen collection is critical for mutation analysis. The aim of the present study was to analyze the *EGFR*, *KRAS* and *BRAF* status in limited cytological material. To the best of our knowledge, this is the first time that the quantitative scale of tumor cells and the percentage of tumor cells in cytological material were evaluated at the early stages of pathomorphological material qualification for *EGFR*, *KRAS* and *BRAF* mutation analysis. Our results revealed that even 100–1,000 tumor cells from fine needle aspiration (FNA) samples provided reliable results of mutation analysis when sensitive real-time polymerase chain reaction (PCR) methods were used. *EGFR* mutations were detected in 10% (7/71) and *KRAS* mutations were detected in 35% (19/54) of the lung adenocarcinoma cases. In addition, we reported the most common inhibiting mutation (p.T790M) found in coexistence with p.L858R in an FNA sample from a patient, for whom short-term improvement after erlotinib treatment was observed before further progression of the disease. Subsequently, mutual exclusion of *EGFR* and *KRAS* mutations was observed. Cytological samples with a small number of tumor cells obtained via FNA, endobronchial ultrasound (EBUS)-transbronchial needle aspiration (TBNA) or brushing are suggested to be used for diagnostic purposes after careful selection by cytopathologists and analysis using a validated, sensitive real-time PCR method.

## Introduction

Selection of patients based on either epidermal growth factor receptor (*EGFR)* mutations or clinical characteristics appears to be an effective approach to optimize EGFR-tyrosine kinase inhibitor (TKI) treatment for chemotherapy-pretreated non-small cell lung cancer (NSCLC) patients (1). In multivariate analyses, *EGFR* mutations found in female East Asian never-smokers with adenocarcinoma were associated with an objective response (2). Most of the mutations found in the *EGFR* catalytic domain are located in a frame deletion in exon 19 and in point mutations in exon 21. The mutations alter the ATP/inhibitor binding site, stabilize the binding of drugs or intensify their inhibitory effect (3). However, patients with activating *EGFR* mutations, for whom tumor progression is observed despite treatment based on TKIs, may acquire resistance to gefitinib or erlotinib (4,5). Mutation analysis employing cytological material sampled using fine needle aspiration (FNA) or cell block of the pleural fluid proved advantageous for the detection of inhibiting mutations in such material (6). Material obtained via endobronchial ultrasound (EBUS)-transbronchial needle aspiration (TBNA) may also be useful for detecting *EGFR* mutations in patients with lung adenocarcinoma, since positive results were previously demonstrated in ~1/10 Spanish patients (7,8).

To the best of our knowledge, no prior studies have described a cytological scale and its correlation with the analysis of *EGFR* and *KRAS* mutations studied in cytological samples obtained from a Polish population. Therefore, the aim of the present study was to analyze the *EGFR*, *KRAS* and *BRAF* status in limited cytological material from Polish patients.

## Materials and methods

### Selection and processing of pathomorphological samples

Cytological material from 78 patients with NSCLC was collected at the Department of Tumor Pathology and Pathomorphology, Oncology Center, Franciszek Lukaszczyk Memorial Hospital in Bydgoszcz, Poland. Informed consent for mutation testing was obtained from all the patients.

A total of 78 specimens were obtained following 67 FNA procedures, 7 bronchial brush procedures, 3 EBUS-TBNA procedures and 1 pleural liquid sampling. In the 78 patients, 75 adenocarcinoma subtypes were determined, and all were tested for the presence of *EGFR* mutations and the majority of samples were tested for *KRAS* mutations. Four cytological samples did not pass quality control steps (pathomorphological qualification or qualitative and quantitative DNA analysis). Biopsy samples were also stained with hematoxylin and eosin (H&E) for qualitative and quantitative analysis of tumor cells in the analyzed material (including macrodissection in marked out samples). Representative cytological smears were subjected to molecular oncological and genetic assessment of mutations in the *EGFR* and *KRAS* genes. Two clinical pathomorphologists (W.J. and C.J.), who were unaware of the patient characteristics, examined the cytological smears as follows. In each smear, two elements were evaluated by the number of neoplastic and non-neoplastic nucleated cells, using a subjective method of microscopic counting of 10 neighboring cells as the smallest virtual ‘decimal cell group’, dispersed uniquely within each tissue section, then 10-folded to the ‘100-fold’ and ‘1,000-fold’ cell groups in the two following steps, yielding the ‘10-fold’, ‘100-fold’ or ‘1,000-fold’ cell measures, respectively, to perform an approximate cell number estimation, when needed, with an accuracy of ~10%. Qualification of cytological material for molecular analysis was based on the following quantitative scale (QS): C1+, ≤100 tumor cells; C2+, >100–1,000 tumor cells; C3+, >1,000–5,000 tumor cells; C4+, >5,000–10,000 tumor cells; C5+, >10,000 (countless) tumor cells) (Table I). Two samples were not qualified due to the small number of cells (no more than 20 tumor cells) (Table II). The percentage of tumor cells (PTCs) was analyzed based on the number of neoplastic cells compared to all nucleated cells in the cytological specimens.

DNA isolation from biopsy smears (after H&E evaluation and image acquisition) was conducted by immersing cytological samples in xylene and by incubation at room temperature overnight or until cover slips were removed (up to 5 days). Once cover slips were taken off, the samples were incubated for 5 min in 100% ethanol, and then washed with 70% ethanol to rehydrate the tissue before the cells were scraped with a sterile scalpel into a tube. Subsequently, DNA was isolated using the QIAamp DNA FFPE tissue kit.

All isolation procedures were performed according to the manufacturer’s instructions. DNA was subjected to qualitative and quantitative analysis (NanoDrop; Thermo Scientific) and DNA isolates with an A260/A280 ratio ranging from 1.8 to 2.0 were tested for the presence of mutations in the *EGFR* and *KRAS* genes.

### Detection of EGFR, KRAS and BRAF mutations

All 29 *EGFR* mutations in exons 18, 19, 20 and 21 were evaluated via the real-time polymerase chain reaction (PCR) methodology using mutation-specific oligonucleotides (EntroGen). A DNA quantity of 600–650 ng was adequate for the detection of 29 mutations in the samples of interest; however, in a few cases the total amount of DNA was reduced to 250 ng for the detection of the 29 mutations due to the high quality of DNA, even though a lower DNA yield was obtained from biopsy samples. Such amount of DNA was in accordance with the instructions for the detection of the 29 *EGFR* mutations, in which the recommended minimum DNA quantity was 50 ng. Mutation analysis was identical to the manufacturer’s protocol for *EGFR* mutation analysis using real-time PCR (EntroGen) performed using LightCycler^®^ 480 II (Roche). For the purpose of validation, direct Sanger sequencing was performed, followed by real-time PCR of the most common mutations in exons 19 and 21. This assay reports the presence of the following *EGFR* mutations: 3 mutations in codon 719 in exon 18; 19 deletions in exon 19; 3 insertions, as well as p.T790M and p.S768I mutations in exon 20; p.L858R and p.L861Q mutations in exon 21. Each analysis was initiated by checking the endogenous control amplification plot of every sample (VIC detector). When endogenous Ctrl CT ranged from 22 to 32, FAM detector was used. When no FAM signal was present, the sample did not contain the selected mutation. Normal positive CT values (mutation detected) may range from 20 to 37. When the CT value was >38, the reaction alone or the entire procedure including DNA isolation was repeated. The *EGFR* mutation assay was established to have an analytical sensitivity of 1% based on dilution studies prepared by EntroGen.

In order to investigate the mutation status in codons 12 and 13 of the *KRAS* gene in 54 cytological samples, HybProbe and melting curve analysis (LightMix^®^ Diagnostic kits; TIB MolBiol) were performed. LightCycler^®^ FastStart DNA Master HybProbe was prepared according to the manufacturer’s instructions, along with the controls and the CTRL, LOW and HIGH reactions with the tested DNA. This assay reports the presence of *KRAS* mutations located in codons 12 and 13: c.34G>C, c.34G>A, c.34G>T, c.35G>A, c.35G>T, c.35G>C, c.37G>T and c.38G>A. Following real-time PCR analyses, melting curve analysis was performed according to the following protocol: the 13C reaction exhibits a peak at ~56°C, the 12C reaction exhibits a peak at ~68–70°C, the NTC reaction exhibits baseline value, the WT reaction exhibits a peak at ~64–65°C, the cWT reaction exhibits baseline value or a maximum of 10% value of the WT reaction signal. Reaction for a sample was analyzed when the control reaction met specific criteria. Wild-type was reported when a sample displayed no peak in the HIGH reaction and the LOW reaction displayed a wild-type-specific peak at the same time. The CTRL reaction also had to demonstrate a wild-type-specific peak. A mutation was reported when a sample displayed a clear peak in the HIGH reaction. The LOW reaction might return the same result or display two peaks corresponding to the wild-type and mutation. A mutation was also reported when a sample returned no peak in the HIGH reaction but had a clear peak at any temperature between 50 and 65°C in the LOW reaction. The CTRL reaction had to show a wild-type-specific peak or display two peaks corresponding to the wild-type and mutation.

Selected samples were confirmed using additional methods: *EGFR* mutation-positive samples via Sanger sequencing (ABI Prism 3130xl Genetic Analyzer), and *KRAS*-*BRAF* mutation-positive samples via Strip Assay, which also allowed the analysis of *BRAF* status in codon 600 (ViennaLab Diagnostics GmbH) according to the manufacturer’s instructions (30).

## Results

### Characteristics of the patients and specimens

#### Clinical information

Clinical characteristics, such as gender, and procedures used for cytological material collection are provided in Table III in correlation with the *EGFR*, *KRAS* and *BRAF* c.1799T>A (V600E) mutation status. Generally, 100% of the patients were Caucasians, among whom most of the *EGFR* mutations were observed in women (85.7%), while most of the *KRAS* mutations were observed in men (65%). *EGFR*, *KRAS* and *BRAF* mutations were detected in material collected using FNA (47 patients), brushing procedure (4 patients) and EBUS-TBNA (3 patients).

#### Specimen evaluation

Every cytological sample that qualified for *EGFR* mutation analysis was carefully analyzed by pathomorphologists at the Oncology Center, Franciszek Lukaszczyk Memorial Hospital. Pathomorphologists selected representative biopsy samples among several collected in each case for further molecular analysis. In order to determine whether mutations could be detected in the cytological material with a small number of tumor cells, identification was performed with samples meeting the following criteria: i) the sample was identified as adenocarcinoma, and ii) the pathological material was available for PTCs and QS in tumor analysis prior to DNA isolation. The real-time PCR methodology was validated using mutated and WT *EGFR* samples previously confirmed by sequencing. Some cytological specimens gave a low yield of genomic DNA extraction but their quality was high enough to perform mutation analysis. All samples were evaluated for several parameters, such as PTCs and QS (Table IV).

All detected mutations were derived from specimens with >1,000 tumor cells. One EGFR mutation in exon 21 was detected in cytological material that qualified for the C3 group with >1,000 tumor cells. Another 4 mutations were detected in specimens that qualified for the C4 group (with >5,000 tumor cells) and 2 mutations qualified for the C5 group (with countless tumor cells). No mutations were detected in C1 and C2 specimens (Table I).

#### EGFR mutation analysis

Among the 75 samples that qualified for evaluation of the presence of *EGFR* mutations in exons 18, 19, 20 and 21 in adenocarcinoma cytological samples, 4 samples did not qualified for molecular analysis since few tumor cells were found in the cytological material (<20) or due to the fact that the low yield and poor quality of the extracted DNA material prevented the use of real-time PCR (Table II).

In the 71 samples analyzed, 7 mutations were detected, mostly regarding a deletion in exon 19, followed by substitutions in exon 21 and a single insertion in exon 20, while 1 sample carried two mutations of inhibiting *EGFR* c.2369C>T (T790M) (Fig. 2B) and activating *EGFR* c.2573T>G (L858R) types in exon 21 (Fig. 2A and Table II). Six *EGFR* mutations were found in the cytological material collected via the FNA procedure and 1 deletion in exon 19 was found in the material obtained using brushing (Table III).

**Figure 1:**
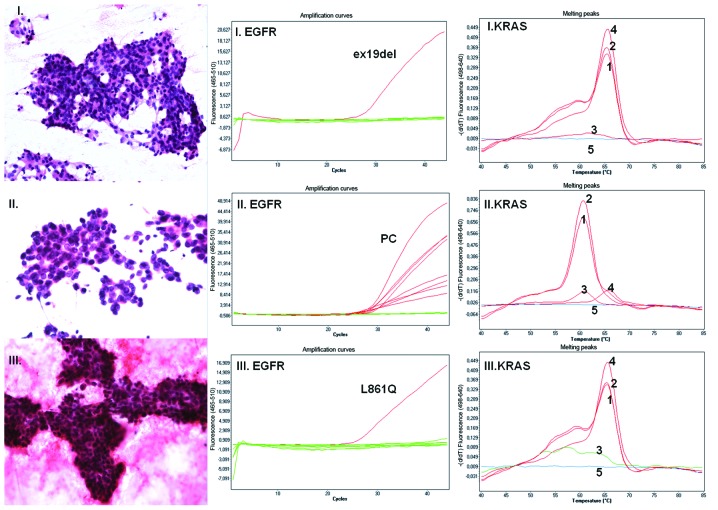
Lung adenocarcinoma. (I–III) Cytology of lung adenocarcinoma following H&E staining (magnification, ×200, ×400 and ×200 for samples I, II and III, respectively). (I–III EGFR) EGFR status detected using mutation-specific oligonucleotides. Green lines represent baseline value with no mutation detected in exons 18, 19, 20 and 21; red lines represent amplification curves as follows: (I. EGFR) *EGFR* exon 19 deletion detected in sample I; (II. EGFR) all positive controls in detection of 29 mutations in sample II, no mutation detected in sample II; (III. EGFR) detection of *EGFR* c.2582T>A (L861Q) in sample III. (I–III KRAS) KRAS status detection. Nos. 1–5 represent melting curves for the CTRL reaction: 1, LOW reaction; 2, HIGH reaction; 3; and for the controls: 4, WT and 5, NTC. Melting curve analysis detected *KRAS* WT (I. KRAS), *KRAS* c.35G>A (G12D) (II. KRAS) and *KRAS* WT (III. KRAS).

The real-time PCR method allowed detection of an *EGFR* c.2582T>A (L861Q) mutation in cytological material with the number of tumor cells ranging from 1,000 to 5,000 (QS=C3+, PTS=95%), obtained using BAC/CT (Fig. 1III). However, most of the mutations were detected with the minimum number of adenocarcinoma cells exceeding 5,000. The *EGFR* mutation detection rate was 10%, taking into consideration the tested cytological samples with a minimum of 1,000 tumor cells (Tables I and IV).

#### KRAS and BRAF mutation analysis

**Figure 2:**
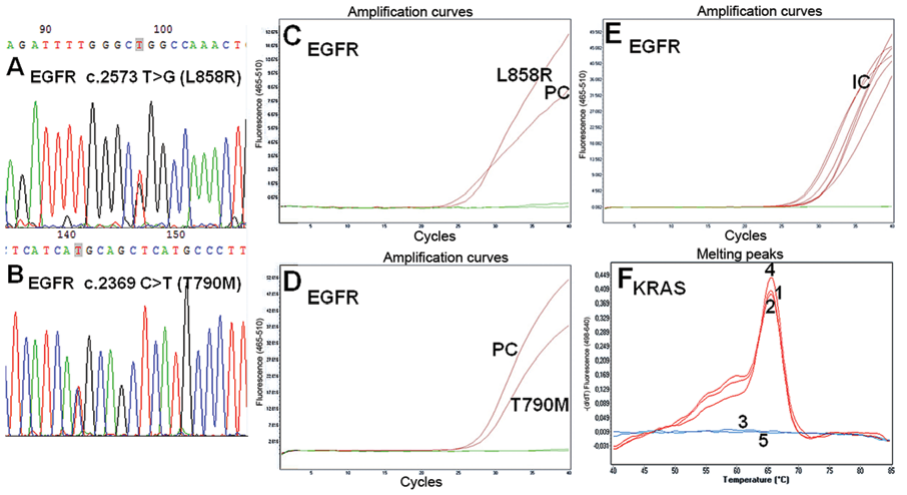
(A and B) Analysis of activating and inhibiting *EGFR* mutations using Sanger sequencing and (C–F) real-time PCR analysis of sample 56. (A and B) Chromatograms presenting *EGFR* substitutions: c.2573 T>G (L858R) and c.2369 C>T (T790M) in *EGFR* (sample 56), respectively. (C and D) Amplification curve for sample 56 with *EGFR* c.2573 T>G (L858R) and c.2369 C>T (T790M) mutations (red lines), respectively. PC, positive control. (E) Internal controls (IC) measuring DNA load of sample 56. (F) Detection of *KRAS* mutations using melting curve analysis. Lack of *KRAS* mutations in exons 12 and 13 in sample 56. 1, CTRL reaction; 2, LOW reaction; 3, HIGH reaction (baseline) and the controls: 4, WT reaction; and 5, NTC reaction.

Out of the 71 samples qualified for real-time PCR analysis and tested for *EGFR* mutations (Table II), only 54 samples were selected for *KRAS* mutation analysis (Table III). Seventeen samples were not used for *KRAS* analysis due to the limited quantity of DNA obtained via extraction from the cytological material, which was depleted for *EGFR* mutation analysis. The presence or absence of the 10th most common *KRAS* mutation in codons 12 and 13 was also determined. We found 11 *KRAS* c.34G>T (G12C), 4 *KRAS* c.35G>T (G12V), 2 *KRAS* c.35G>A (G12D) mutations (Fig. 1II) and a single mutation in codon 13, *KRAS* c.38G>A (G13D). Every *KRAS* mutation analysis was confirmed using different PCR methods: HRM analysis (TIB MolBiol) and biotinylated sequences detected using streptavidin-alkaline phosphatase (ViennaLab Diagnostics GmbH). The *BRAF* c.1799T>A (V600E) mutation was analyzed in only 19 samples (due to limited cytological material), in which a *KRAS* mutation was detected (Table IV). Notably, the real-time PCR method allowed detection of the *KRAS* c.35G>T (G12V) mutation in cytological material characterized by QS=C2+ and PTS=80% (Table IV). No cases of concurrent presence of *KRAS* and *EGFR* mutations were observed in the same tumor sample (Table III). The majority of samples (n=28) were *EGFR* wild-type and *KRAS* wild-type. Seven patients with an *EGFR* mutation lacked *KRAS* mutations in codons 12 and 13 (Fig. 2F) and vice versa. One patient with wild-type *EGFR* and a *KRAS* mutation in codon 12 (G12C) had a mutation in *BRAF* c.1799T>A (V600E).

## Discussion

The presence of an *EGFR* mutation in the tyrosine kinase domain constitutes an important predictive factor for the response to treatment with TKIs (9). Therefore, it is crucial to analyze the *EGFR* mutation status using the most reliable method, which permits detection of activating and inhibiting *EGFR* mutations.

In the real-time PCR and new generation sequencing era, small quantities of input material may be used for the analysis of numerous mutations. However, the heterogeneous character of tumors should not be underestimated. Therefore, quantitative estimation of cytological specimens should be performed in the preanalytical step, in order to determine mutations in as many tumor cells as possible. The findings of the present study suggest that *EGFR* mutations are detected in DNA isolated from heterogeneous cytological material containing ≥1,001 tumor cells. Therefore, the *EGFR* mutation analysis in cytological specimens may be performed as a routine evaluation in patients with inoperable NSCLC. In contrast, unequal cytological sampling could be potentially misleading. Any *EGFR* WT result should be carefully assessed as it might reflect sampling bias, particularly when the analysis was performed using DNA isolated from a small number of tumor cells (21–1,000). Taking into consideration the histological heterogeneity of NSCLC, genetic differentiation implied as local divergence of mutation status within a tumor may also be possible. Thus, the fact that cytological samples become less representative with a decreasing number of tumor cells cannot be excluded. In such a case, potential *EGFR* WT results obtained following analysis of cytological specimens with a small number of tumor cells might be considered as potentially containing false negatives, and different results might be obtained following the analysis of an additional FNA sample from the same patient. Cytological material may be analyzed using smeared slides or cytological cell blocks. This leads to the serious drawback of the time-consuming digital archiving of smear slides which is ‘sacrificed’ for DNA isolation.

Cytological smears constitute material more difficult to handle when compared to paraffin-embedded tissues; therefore, it is highly important to use well-validated, sensitive methods which do not provide false-positive results, and also decrease any discrepancies and false-negative results of the *EGFR* mutation status analysis, when DNA material derived from cytological samples is limited. Currently, different methods of *EGFR* status assessment are used, ranging from immunocytochemistry (10) and qualitative or quantitative PCR methods (11) to sequencing (including either Sanger or new generation sequencing methods). Taking into consideration that in most cases of inoperable NSCLC the existing cytological material is critical, we evaluated the real-time PCR detection of *EGFR* mutations in exons 18, 19, 20 and 21 of adenocarcinoma cells obtained via FNA, EBUS, TBNA, brushing procedures, and thoracentesis. In the present study, we found that 10% of the 77 analyzed adenocarcinoma samples harbored *EGFR* mutations, 35% of which carried *KRAS* mutations, while 51% of the 54 samples analyzed for *KRAS* mutations were negative regarding both mutation types. *KRAS* mutation results were moderately high for the detection of *KRAS* mutations with cytomorphological features of adenocarcinomas (12,13), but are in accordance with the recently reported prevalence of NSCLC patients with *KRAS* mutations (27%) detected using COLD-PCR (14) and 36.9% *KRAS* mutations detected in malignant pleural effusion of lung adenocarcinoma in a Dutch population (15).

*EGFR* mutations detected using an assay based on amplifying mutant-specific sequences (EGFR-RT52; EntroGen) were confirmed by Sanger sequencing. *KRAS* mutations were detected using high melting temperature (LightMix^®^ kit; TIB MolBiol), a method known to provide false-positive results (16). Therefore all samples with detected *KRAS* mutations were verified using another method, KRAS Strip Assay. In fact, in the case of the two samples, the use of high melting temperature yielded borderline results [c.35G>A (G12D)], while following verification with a second method (ViennaLab Diagnostics GmbH), the samples were evaluated as KRAS-WT (data not shown). It should be taken into consideration that direct sequencing is not always sensitive enough to detect mutant DNA (17), and that high-resolution melting analysis may also give false-negative results (18). Notably, two false-negative results were found in cytological material with a small proportion of tumor cells, indicating that cytologists should not only distinguish benign and malignant samples, but they should also conduct proper qualification of the material for molecular analysis (18). The real-time PCR analysis of *EGFR* and *KRAS* mutations conducted in the present study, led to identification of *EGFR* c.2582T>A (L861Q) and *KRAS* c.35G>T (G12V), detected in 1,000–5,000 and 100–1,000 tumor cells, respectively. Thus, our results confirm that cytological samples characterized by QS=C3+ or even C2+ should not be evaluated as inadequate for *EGFR* mutation analysis using real-time PCR, but should be carefully selected by a cytopathologist.

Mutations in *EGFR* and *KRAS* detected in tumors embedded in paraffin blocks and fresh-frozen tumor specimens appear to be mutually exclusive (19). Our results obtained following analysis of cytological material are consistent with this observation (Table I and Fig. 1). Usually, *EGFR* mutations are characteristic of tumors in the non-smoker groups of patients, particularly among Asian women, while *KRAS* mutations are often detected in smoking-associated cancer types. Therefore, *KRAS* mutations have been suggested to constitute a primary mechanism of resistance to gefitinib or erlotinib in lung adenocarcinoma. This finding does not clarify whether this insensitivity is due to the presence of the mutated *KRAS* or the absence of the mutated *EGFR*(20). In contrast, the acquired resistance to TKIs has been studied to a great extent, particularly due to mutations in exon 20. The most frequent TKI mutation is *EGFR* c.2369C>T (T790M) (20), followed by insertions in exon 20 which may render the epidermal receptor approximately 100-fold less sensitive to erlotinib or gefitinib (21). Concerning the findings of the present study, 1 adenocarcinoma patient was found to carry both *EGFR* activating c.2573T>G and inhibiting c.2369C>T mutations following analysis of material isolated via the FNA procedure, indicating that the mutations were of activating and inhibiting types, respectively. The structure of the wild-type *EGFR* kinase domain has been published in both active and inactive conformations, while its crystal structure has been reported alone and in complex with erlotinib (22). Along with the discovery of the *EGFR* c.2573T>G (L858R) EGFR mutant crystal structure, which is TKI-sensitive, it was indicated that the substitution of arginine for leucine at position 858 activates the kinase. Comparison of the fold activity between the wild-type and the L858R mutant enzyme demonstrated an approximately 50-fold higher activity of the mutant conformation (23). Further studies of gefitinib revealed that this 4-anilinoquinazoline inhibitor, structurally similar to erlotinib, binds the *EGFR* c.2573T>G (L858R) mutant with a 20-fold higher affinity compared to the wild-type enzyme. This higher affinity for the *EGFR* c.2573T>G (L858R) mutant was explained by tighter binding to the active conformation of the kinase compared to the inactive conformation (23). In contrast, some patients were reported to become resistant following TKI treatment due to mutation in the ‘gatekeeper’ residue of threonine 790 (24,25). Another inhibiting mutation in exon 21 is the A→G change, which leads to the substitution of alanine for threonine at position 854 (5). Both mutations, the activating *EGFR* c.2573T>G (L858R) and the inhibiting *EGFR* c.2369C>T (T790M) were found in our cytological assessment.

The cytological samples of the present study obtained via FNA, were found to carry both mutations *EGFR* c.2573T>G and c.2369C>T (L858R/T790M), demonstrating the advantage of the FNA procedure and the ability of performing serial sampling of a given tumor to assess the efficacy of targeted therapy or identify genetic shifts of adenocarcinoma with EGFR mutations (3). This recommendation does not exclude patients from targeted therapies, since patients with the *EGFR* c.2369C>T (T790M) mutation of EGFR might still respond to erlotinib after gefitinib treatment (6). In fact, the patient with *EGFR* c.2573T>G (L858R) and *EGFR* c.2369C>T (T790M) was treated with erlotinib and a short-term improvement was observed. However, 7 months after the detection of the L858R/T790M mutation, a CT scan of the chest and abdomen revealed metastases to both lung and liver, resulting in the death of the patient.

According to a recent anticancer drug discovery using a high-throughput cell-based assay, inhibitors of the L858R/T790M mutant EGFR pathway were identified after screening 60,000 compounds (26). Those findings may also allow classification of novel inhibitors which suppress mutant *EGFR* c.2369C>T (T790M) signaling (26). An additional study on a classical protein kinase C inhibitor, revealed high potency against the mutated *EGFR* and significantly reduced tumor growth in an *in vivo* xenograft model employing an EGFR-mutant NSCLC cell line containing the *EGFR* c.2369C>T (T790M) mutation (27). Moreover, novel EGFR TKIs which bind irreversibly to EGFR-TK and form covalent cross-links with EGFR, such as afatinib (BIBW2992), have been demonstrated to be active against tumors resistant to reversible EGFR TKI (28,29).

In conclusion, cytological material is useful for the assessment of the *EGFR* mutation status for assessment of personalized therapy with EGFR-TKIs. We demonstrated that the real-time PCR methods used here may allow detection of activating and inhibiting mutations. Furthermore, in cytology material with >1,000 tumor cells in samples from a Polish Caucasian population, there was a frequency of 10% *EGFR* mutations, while in samples with a minimum of 5,000 tumor cells the frequency was 12,24%. Our data suggest the presence of false-negative cases within the *EGFR* WT results, obtained from the cytological specimens with a small number of tumor cells (even up to 1,000). Molecular EGFR and KRAS testing of cytological material could provide also further information to oncologists and pathomorphologists since the presence of *EGFR* mutations is mutually exclusive of *EML4-ALK* transcript and low percentage of KRAS mutation (11%) is found in NSCLC specimens carrying the *EML4-ALK* transcripts (31).

## Figures and Tables

**Table I:** Quantitative scale (QS) and sample numbers of detected *EGFR* and *KRAS* mutations.

QS	No. of tumor cells	No. of *EGFR* mutations detected	No. of *KRAS* mutations detected
C1+	>20–100	None	None
C2+	>100–1,000	None	1
C3+	>1,000–5,000	1	7
C4+	>5,000–10,000	4	8
C5+	>10,000 (countless)	2	3

**Table II:** *EGFR* mutation status in the studied lung adenocarcinomas.

EGFR	Exon	No. of patients	Percentage of patients	Mutation (presence or absence)/no. of tests performed
*EGFR* wild-type	18, 19, 20 and 21	64	64/71 (90%)	**64/71 (90%)**
Exon 19 deletion	19	4	4/7 (57.1%)	**7/71 (10%)**
Exon 20 insertion	20	1	1/7 (14.3%)	
c.2582T>A (L861Q)	21	1	1/7 (14.3%)	
c.2369C>T (T790M)/c.2573T>G (L858R)	18 and 21	1	1/7 (14.3%)	
No. of disqualified analyses	-	4	4/75 (5.3%)	-

**Table III:** Clinicopathological characteristics of 54 lung adenocarcinomas according to the *EGFR* and *KRAS* mutation status.

	Gender, n		Procedure, n		
		
Group	Female	Male	FNA	EBUS and TBNA	Brushing
EGFR^+^/KRAS^−^ (n=7)	6	1	6	0	1
EGFR^−^/KRAS^+^/BRAF^−^ (n=18)	5	13	16	1	1
EGFR^−^/KRAS^+^/BRAF^+^ (n=1)	0	1	1	0	0
EGFR^−^/KRAS^−^ (n=28)	20	8	24	2	2

FNA, fine needle aspiration; EBUS, endobronchial ultrasound; TBNA, transbronchial needle aspiration.

**Table IV:** *EGFR*, *KRAS* and *BRAF* mutations detected in cytological material qualified for molecular analysis using quantitative scale (QS) and the percentage of tumor cells (PTCs).

Mutation detected	C+ (QS)	% (PTCs)	Procedure
*EGFR* exon 19 deletion	C4+	Unknown	FNA
*EGFR* exon 19 deletion	C4+	80	FNA
*EGFR* exon 19 deletion	C4+	80	FNA
*EGFR* exon 19 deletion	C4+	50	Brushing
*EGFR* exon 20 insertion	C5+	90	FNA
*EGFR* c.2582T>A (L861Q)	C3+	95	FNA
*EGFR* c.2369C>T (T790M) and *EGFR* c.2573T>G (L858R)	C5+	Unknown	FNA
*KRAS* c.34G>T (G12C)	Unknown	Unknown	FNA-CT
*KRAS* c.35G>T (G12V)	C3+	50	TBNA
*KRAS* c.35G>T (G12V)	C3+	Unknown	FNA
*KRAS* c.34G>T (G12C)	C3+	30	Brushing
*KRAS* c.34G>T (G12C)	C3+	60	FNA
*KRAS* c.35G>A (G12D)	C4+	80	FNA
*KRAS* c.34G>T (G12C)	C4+	75	FNA
*KRAS* c.35G>A (G12D)	C4+	80	FNA
*KRAS* c.35G>T (G12V)	C3+	60	FNA
*KRAS* c.34G>T (G12C)	C4+	80	FNA
*KRAS* c.35G>T (G12V)	C2+	80	FNA
*KRAS* c.34G>T (G12C)	C3+	40	Brushing
*KRAS* c.34G>T (G12C)	C5+	90	FNA
*KRAS* c.35G>T (G12V)	C4+	80	FNA
*KRAS* c.34G>T (G12C)	C4+	90	FNA
*KRAS* c.34G>T (G12C)	C5+	80	FNA
*KRAS* c.35G>A (G12D)	C4+	70	FNA
*KRAS* c.34G>T (G12C)	C5+	80	FNA
*KRAS* c.34G>T (G12C)	C4+	80	FNA
*BRAF* c.1799T>A (V600E)	C3+	60	FNA

FNA, fine needle aspiration; CT, computed tomography; TBNA, transbronchial needle aspiration.
